# *In vitro* pharmacokinetics/pharmacodynamics of FL058 (a novel beta-lactamase inhibitor) combined with meropenem against carbapenemase-producing Enterobacterales

**DOI:** 10.3389/fphar.2024.1282480

**Published:** 2024-04-11

**Authors:** Zhiwei Huang, Xingchen Bian, Yi Li, Jiali Hu, Beining Guo, Xinyi Yang, Yi Jin, Shansong Zheng, Xinmei Wang, Cong Gao, Jing Zhang, Xiaojie Wu

**Affiliations:** ^1^ Institute of Antibiotics, Huashan Hospital, Fudan University, Shanghai, China; ^2^ Phase I Clinical Research Center, Huashan Hospital, Fudan University, Shanghai, China; ^3^ Qilu Pharmaceutical Co Ltd, Jinan, China

**Keywords:** beta-lactamase inhibitor, meropenem, *in vitro* model, pharmacokinetics/pharmacodynamics, carbapenemase-producing Enterobacterales

## Abstract

**Objective:** FL058 is a novel beta-lactamase inhibitor with a broad spectrum of activity and a favorable safety profile. The objective of this study was to evaluate pharmacokinetic/pharmacodynamic (PK/PD) relationships for the combination of FL058 and meropenem in an *in vitro* infection model.

**Methods:** By simulating human concentration-time profiles in the *in vitro* model, meropenem combined with FL058 when administered 1 g/0.5 g, 1 g/1 g, 2 g/1 g, and 2 g/2 g q8h by 3-h infusion achieved approximately 2- and 4-log10 kill to KPC/OXA-producing *Klebsiella pneumoniae* and *Escherichia coli*; the combination therapy could not inhibit NDM-producing *K. pneumoniae* but could maintain NDM-producing *E. coli* around a baseline.

**Results:** The PK/PD indexes that best described the bacterial killing from baseline in log10 CFU/mL at 24 h were the percent time of free drug above the minimal inhibitory concentration (MIC) (%fT > MIC, MIC with FL058 at 4 mg/L) for meropenem and the percent time of free drug above 1 mg/L (%fT > 1 mg/L) for FL058. The targets for achieving a static effect and the 1- and 2-log10 kill were 74, 83, and 99 for %fT > MIC of meropenem and 40, 48, and 64 for %fT > 1 mg/L of FL058, respectively. The PK/PD index of %fT > 1 mg/L can provide a basis for evaluating clinical dosing regimens for FL058 combined with meropenem.

**Conclusion:** FL058 combined with meropenem might be a potential treatment for KPC- and/or OXA-48-producing Enterobacterales infection.

## Introduction

The extensive use of antibacterial agents has resulted in rapidly increasing drug-resistant bacteria. Against this backdrop, the detection rate of multidrug-resistant bacteria represented by Gram-negative bacilli is increasing, posing a huge challenge to anti-infective therapy in clinical practice. According to the data from CHINET (www.chinets.com), an antimicrobial surveillance network, the resistance rate of *Klebsiella pneumoniae* to meropenem in tertiary hospitals has increased from 2.9% in 2005 to 24.4% in 2021. For *Escherichia coli*, the resistance rate to meropenem reaches 1.4%–2.1%. The primary mechanism of Enterobacterales resistance to beta-lactam antibiotics is the production of beta-lactamase. Beta-lactamases are grouped into four classes according to the Ambler classification system: Class A (e.g., extended-spectrum beta-lactamases, ESBLs; and *K. pneumoniae* carbapenemases, KPCs), Class B (e.g., New Delhi metallo-beta-lactamases, NDMs), Class C (e.g., AmpC cephalosporinases), and Class D (e.g., oxacillinases, OXAs). A large investigational survey of carbapenem-resistant Enterobacterales (CRE) revealed that KPCs are the most prevalent beta-lactamases and NDMs are the second most prevalent beta-lactamases in *K. pneumoniae* (Wang et al., 2018). In recent years, OXAs have become more common in carbapenem-resistant *K. pneumoniae* (Tangden and Giske, 2015; Yin et al., 2017).

In consideration of the diversity of the above-mentioned beta-lactamases, researchers have paid close attention to the development of novel broad-spectrum beta-lactamase inhibitors (Shlaes, 2013; Bush, 2015; Vanscoy et al., 2016; Bhagwat et al., 2019). Presently, novel beta-lactamase inhibitors of the non-beta-lactam structure have been marketed, including avibactam, relebactam, and vaborbactam. Neither relebactam nor vaborbactam can inhibit Class D beta-lactamases. FL058 is a novel diazabicyclooctane (DBO) beta-lactamase inhibitor with a structure and activity similar to avibactam. It mainly inhibits Class A, Class C, and some Class D beta-lactamases but does not inhibit NDMs (Sharma et al., 2016). An *in vitro* susceptibility study (to be published) showed that, unlike avibactam, FL058 alone had certain inhibitory activity on *E. coli*. Meropenem combined with 4 μg/mL FL058 had a significantly lower minimal inhibitory concentration (MIC) for NDM-producing *E. coli* (MIC_90_ = 0.5 mg/L) and partial inhibitory action on NDM-producing *K. pneumoniae* (MIC_50_ = 0.25 mg/L, MIC_90_ = 4 mg/L). A completed phase I clinical trial showed FL058 had good safety, tolerance, and pharmacokinetic (PK) characteristics (Huang et al., 2023).

*In vitro* pharmacokinetic/pharmacodynamic (PK/PD) models have become important tools for screening dosing regimens for beta-lactam antibiotic/beta-lactamase inhibitor therapies (Macgowan et al., 2016; Vanscoy et al., 2016; Macgowan et al., 2017; Sabet et al., 2018). They can also be used to assess the correlation between exposure to a beta-lactam antibiotic/beta-lactamase inhibitor and changes in the colony count. The subsequent analysis of the exposure-response relationship, in turn, can support dosage selection. In light of this, this study simulated the clinical dosing regimens for FL058 combined with meropenem in an *in vitro* model to find the best component ratio of the two drugs and the best PK/PD index and targets for the two-drug combination therapy.

## Materials and methods

### Strains

The study used eight strains of KPC-2-, NDM-1-, or OXA-48-producing Enterobacterales (six strains of *K. pneumoniae* and two strains of *E. coli*), which were provided by Microbiology Division, Institute of Antibiotics, Huashan Hospital, Fudan University.

### Compounds

FL058 (Qilu Pharmaceutical Co., Ltd.; Batch No.: B0220E01; purity: 98.9%) and meropenem (Sumitomo Dainippon Pharma; Batch Nos.: 2329C, 2407C, and 2408C).

### MICs

The MICs of meropenem alone, FL058 alone, and two-drug combination therapy (FL058 concentration: fixed concentration of 4 μg/mL; concentration ratio of meropenem: FL058 = 1:1, 2:1, and 4:1) were determined by broth microdilution in accordance with the standard of Clinical and Laboratory Standards Institute (CLSI). Briefly, 50 μL of broth containing 5 × 10^5^ CFU/mL bacteria were mixed with 50 μL of the drug solution in a 96-well plate in duplicate. The drug solution was either subjected to gradient dilution by a factor of two, or the dosage of FL058 was fixed at 4 μg/mL. MIC values were read with the naked eye within 16–20 h.

### *In vitro* PK simulation

An *in vitro* PK/PD model was established to simulate drug concentration in human plasma (Liang et al., 2011; Liu et al., 2016; Bian et al., 2019). Drug-containing or blank culture media were pumped into the central compartment with a peristaltic pump under segmented control by WinLIN 3.2 (Cole-Parmer) to simulate the intravenous instillation of the drug or drug elimination process. In the study, the following PK characteristics of meropenem in healthy Chinese subjects were simulated: distribution-phase half-life (t_1/2,α_), 0.37 h; elimination-phase half-life (t_1/2,β_), 1.3 h; maximum concentration (C_max_), 23.0 mg/L (1 g of meropenem, infusion 2 h); and peak time (T_max_) at the end of infusion (Zhao et al., 2004). A Phase I clinical trial showed that FL058 in healthy Chinese subjects had an elimination half-life close to that of meropenem (Huang et al., 2003). Therefore, the concentration–time curve of FL058 was simulated similarly to that of the meropenem dosing. In the study, the C_max_ of FL058 was 42.7 mg/L (1 g of FL058, infusion 2 h). The simulation of the dosing regimens of FL058 combined with meropenem based on the *in vitro* PK/PD model is shown in Table 1. Culture media samples were collected before dosing and after 0.5 h, 1 h, 1.5 h, 2 h (immediately at the end of the instillation), 2.5 h, 3 h, 4 h, 6 h, 8 h, 10 h, 16 h, 18 h, and 24 h and stored at −70°C. The concentrations of meropenem and FL058 were determined by liquid chromatography-tandem mass spectrometry (LC-MS/MS) with an Atlantis®T3, 3 μm, 2.1 × 100 mm column, using 0.1% formic acid-8 mM sodium acetate in water and 0.1% formic acid–8 mM sodium acetate in 90% acetonitrile as mobile phases. The flow rate of the mobile phases was 0.6 mL/min. Both meropenem and FL058 had a linearity range of 0.05–50.0 mg/L.

**TABLE 1:** Dosing regimens of FL058 in combination with meropenem.

(h)	Meropenem (g)	FL058 (g)
q8	1	0.125
		0.25
		0.5
		1
	2	0.25
		0.5
		1
		2
q12	1.5	0.75
	3	1.5
q24	3	1.5
	6	3

Infusion 2 h. The label of meropenem suggests a dose of 1 g or 2 g for adults. The dosing regimens of FL058 were formulated to maintain proportionality with the meropenem dosage.

### Antibacterial effects

A single colony on an agar plate was selected and inoculated into the broth for culture overnight on the shaker, and then the bacterial culture liquid was diluted to ca. 0.5 McFarland Standard (ca. 10^8^ CFU/mL); 1.8 mL of the dilution was injected into the central compartment (180 mL) and precultured for 1 h before being inoculated into the *in vitro* model. A 1 mL aliquot of the bacterial culture fluid was sampled from the central compartment at preset time points (before dosing and 1 h, 2 h, 4 h, 6 h, 8 h, 10 h, 16 h, 18 h, and 24 h after dosing) with a sterile injector; 10 μL of the diluted solution or the bulk bacterial culture fluid was placed on agar plates for 18–24 h of culture, then subjected to colony counting. The lower limit of colony count was 100 CFU/mL.

### PK and PK/PD analyses

The measured concentration data were fitted to targeted concentration data by linear fitting (weighed *1/Y*) with the Linear module in Phoenix WinNonlin (version 8.1, Certara) to determine their relationship. The PK parameters of meropenem and FL058 in the *in vitro* model were calculated using the NCA and PK modules in Phoenix WinNonlin, including area under the concentration–time curve from 0 to 24 h (*f*AUC_24_, *f* denotes free drug), maximum concentration (*f*C_max_), *f*%T > C_T_ (threshold concentration, C_T_: 0.25, 1, and 4 mg/L), and *f*%T > MIC. The correlation between the PK/PD indexes and the change from colony count baseline at 24 h was analyzed with the sigmoid E_max_ model in the PD module in Phoenix WinNonlin. The PK/PD indexes selected for FL058 were *f*AUC_24_ and *f*%T > C_T_ in a fixed dosage regimen of meropenem (q8h by 2-h infusion). The PK/PD indexes selected for meropenem were *f*AUC_24_/MIC, *f*C_max_/MIC, and *f*%T > MIC (MIC for FL058 at a fixed concentration of 4 mg/L) in a meropenem-to-FL058 dose ratio of 2:1 (Lepak et al., 2019). Dosing regimens of FL058 and meropenem for PK/PD analysis are shown in Supplementary Table S1. A larger *R* ^2^ and a smaller coefficient of variation indicated a better PK/PD index.

## Results

### Susceptibility testing

The MICs for FL058 and meropenem alone and in combination are shown in Table 2; all strains were resistant to meropenem (MICs ≥8 mg/L). FL058 restored the susceptibility of *K. pneumoniae* and *E. coli* to meropenem, and FL058 monotherapy had certain activity against ATCC BAA-1705 and ATCC BAA-2452. The MICs were apparently lower when the concentration of FL058 was fixed at 4 μg/mL, at which concentration the MIC of meropenem decreased by a factor of 8–512.

**TABLE 2:** Susceptibility testing results of meropenem alone, FL058 alone, or meropenem combined with FL058 against *K. pneumoniae* and *E. coli*.

	Isolate no.	Type of beta-lactamase	Meropenem	FL058	MIC of meropenem (in combinationcombined with FL058)			
					1:1	2:1	4:1	Meropenem: 4 μg/mL FL058
*K. pneumoniae*	ATCC BAA-1705	KPC-2	16	4	0.5	0.5	1	≤0.06
	17-R1-16	KPC-2, CTX-M-14	>64	>64	2	4	4	0.25
	17-R1-38	KPC-2, CTX-M-14	64	>64	2	2	4	0.5
	18-W45-56	KPC-2, CTX-M-14	>64	64	4	8	8	0.5
	20-W2-70	OXA-48	32	8	4	8	8	1
	17-R1-95	NDM-1	64	32	4	4	8	4
	17-R2-27	NDM-1	64	64	8	8	8	8
*E. coli*	ATCC BAA-2452	NDM-1	32	4	2	4	8	≤0.06
	20-W2-18	KPC-2	>64	32	1	2	2	0.25

### PK simulation

**FIGURE 1:**
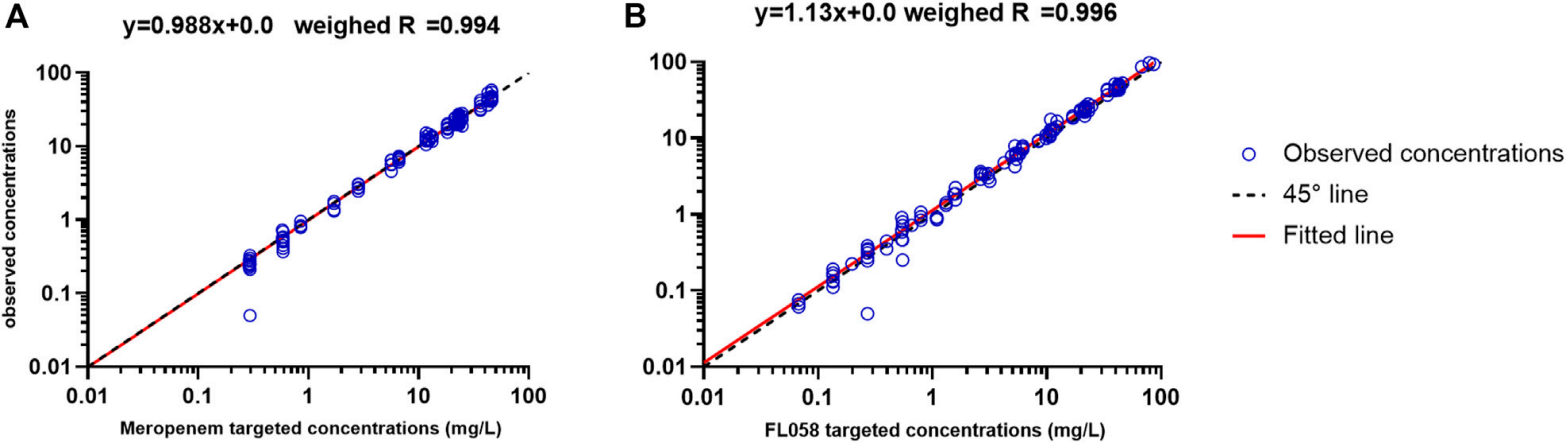
Accuracy of observed concentration of **(A)** meropenem and **(B)** FL058 vs. targeted concentration (open blue circles denote observed concentration points, solid red line denotes the fitted straight line, dashed black line denotes the 45° line).

Figure 1 shows the linear fitting of the observed concentrations to the targeted concentrations of the *in vitro* PK model. Meropenem’s linear fitting equation was *y = 0.988x+0.0*, and weighed R = 0.994. The linear fitting equation for FL058 was *y = 1.13x+0.0*, and weighed R = 0.996, suggesting that the PK profiles in the *in vitro* model can satisfactorily match the PK profiles of meropenem and FL058 in humans.

### Time–killing curves

**FIGURE 2:**
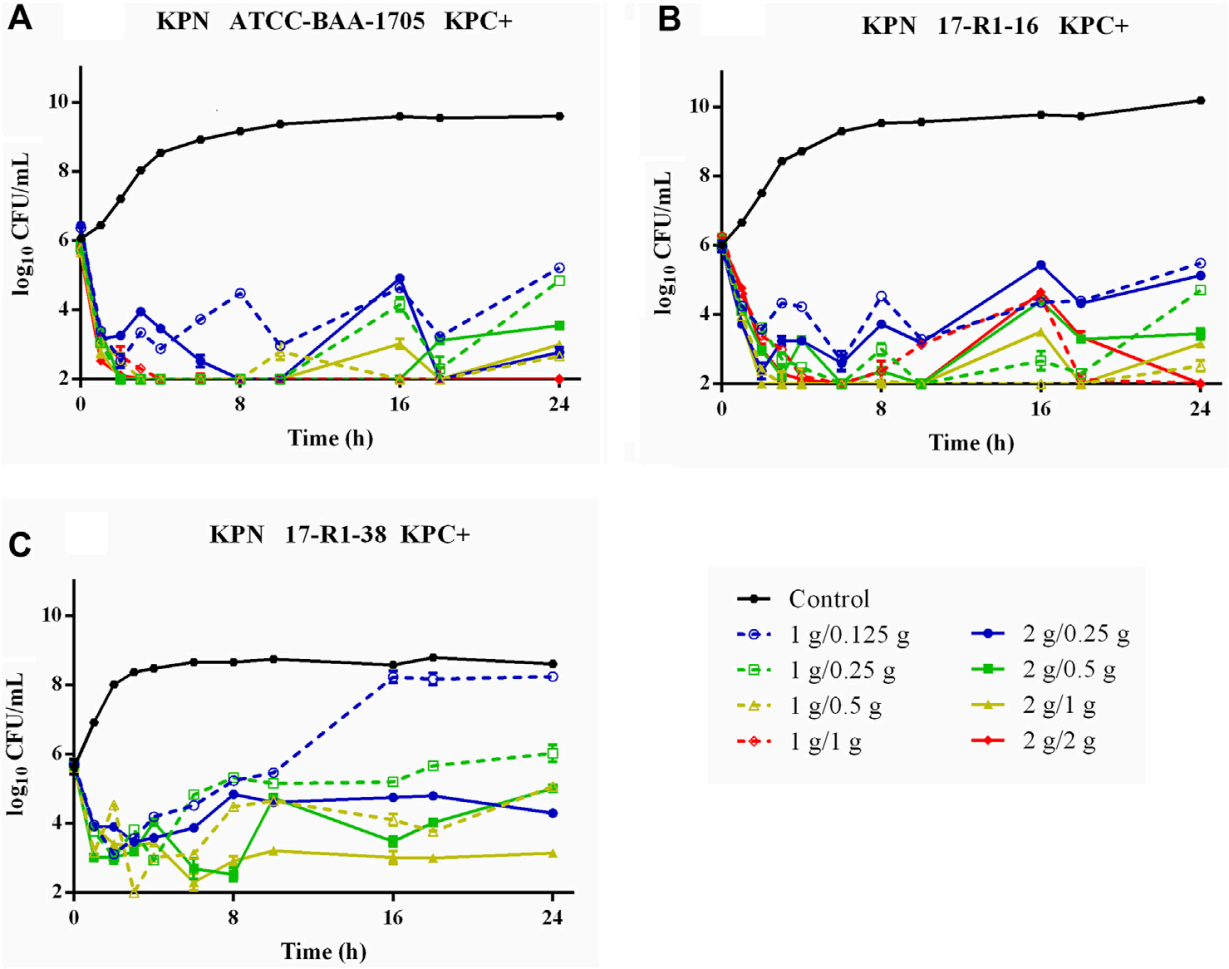
Time–killing curves of meropenem in combination with FL058 q8h by a 2-h infusion against three KPC-producing *K. pneumoniae*. **(A)** *K. pneumoniae* ATCC BAA-1705, **(B)** *K. pneumoniae* 17-R1-16, and **(C)** *K. pneumoniae* 17-R1-38. Tick marks on the *x*-axis are the start of each infusion. KPN: *K. pneumoniae*.

**FIGURE 3:**
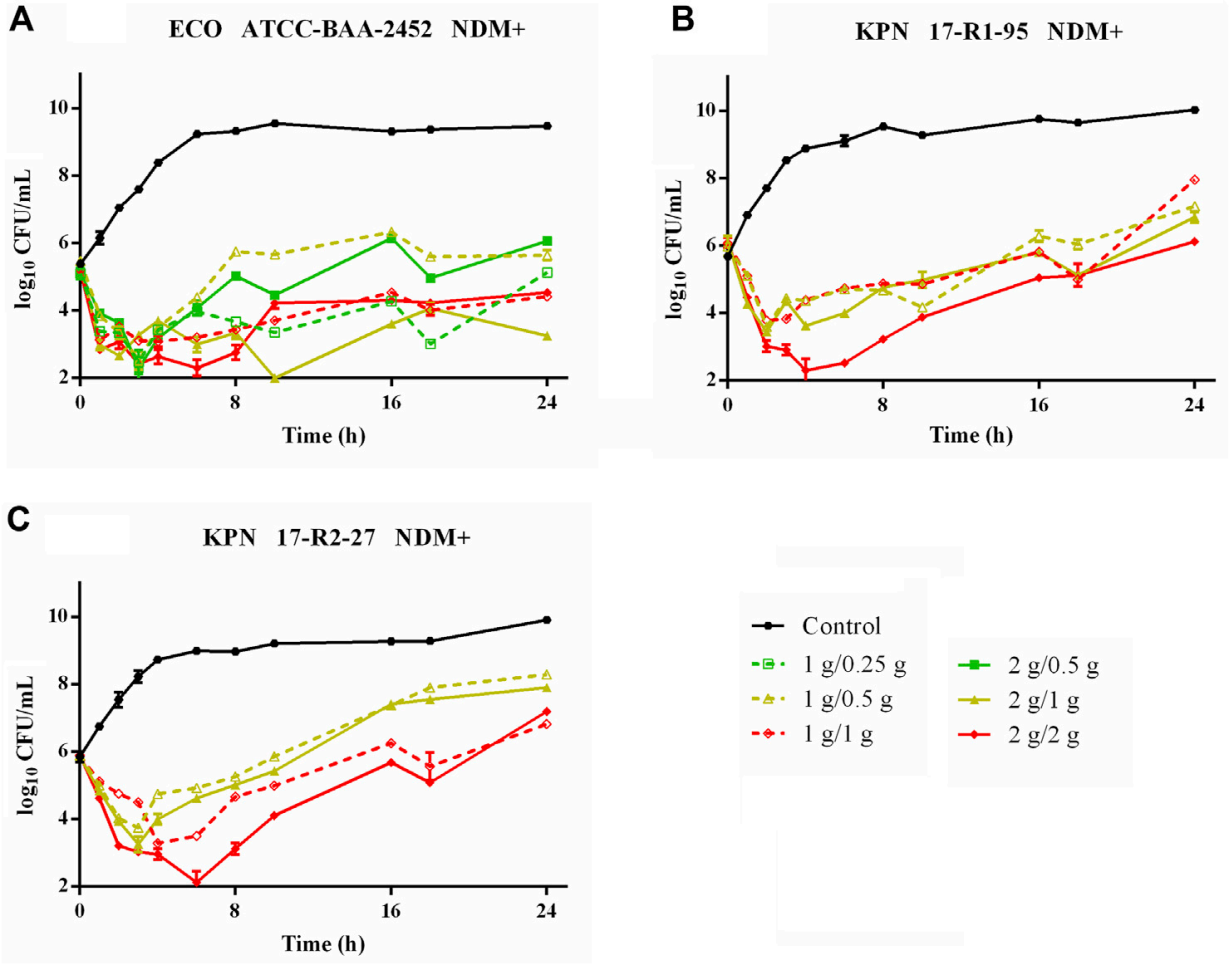
Time–killing curves of meropenem in combination with FL058 q8h by a 2-h infusion against NDM-producing Enterobacterales: **(A)** *E. coli* ATCC BAA-2452, **(B)** *K. pneumoniae* 17-R1-95, and **(C)** *K. pneumoniae* 17-R2-27. Tick marks on the *x*-axis are the start of each infusion. KPN: *Klebsiella pneumoniae*, ECO: *Escherichia coli*.

Time–killing curves of FL058 combined with meropenem (q8h) and no-treatment control groups against *K. pneumoniae* are shown in Figure 2. All dose regimens quickly killed the bacteria in 0–2 h, but at 8 h (the trough concentration point), rebound growth of bacteria was frequently observed. This was most evident with the 1 g/0.125g, 1 g/0.25 g, and 2 g/0.25 g (meropenem/FL058) combinations. Curves showing the killing of two strains of NDM-producing *K. pneumoniae* and one strain of NDM-producing *E. coli* by FL058 combined with meropenem are shown in Figure 3. Four dosage combinations for meropenem/FL058 (1 g/0.5 g, 1 g/1 g, 2 g/1 g, and 2 g/2 g) had a killing effect on two strains of NDM-producing *K. pneumoniae* (17-R1-95 and 17-R2-27) in 2 h, but the bacteria resumed growth starting from 3 to 4 h postdose, and the growth continued. By 24 h, the bacterial load exceeded the baseline. In 3 h, all six dosage regimens for FL058 combined with meropenem killed approximately 2-log_10_ CFU/mL of NDM-producing *E. coli* ATCC BAA-2452. The bacterium resumed growth starting from 4 to 8 h, but these combinations maintained an inhibitory effect in 8–24 h. Time–killing curves of FL058 combined with meropenem against KPC- or OXA-producing *K. pneumoniae* and KPC-producing *E. coli* are shown in Supplementary Figure S1.

### PK/PD analysis

**FIGURE 4:**
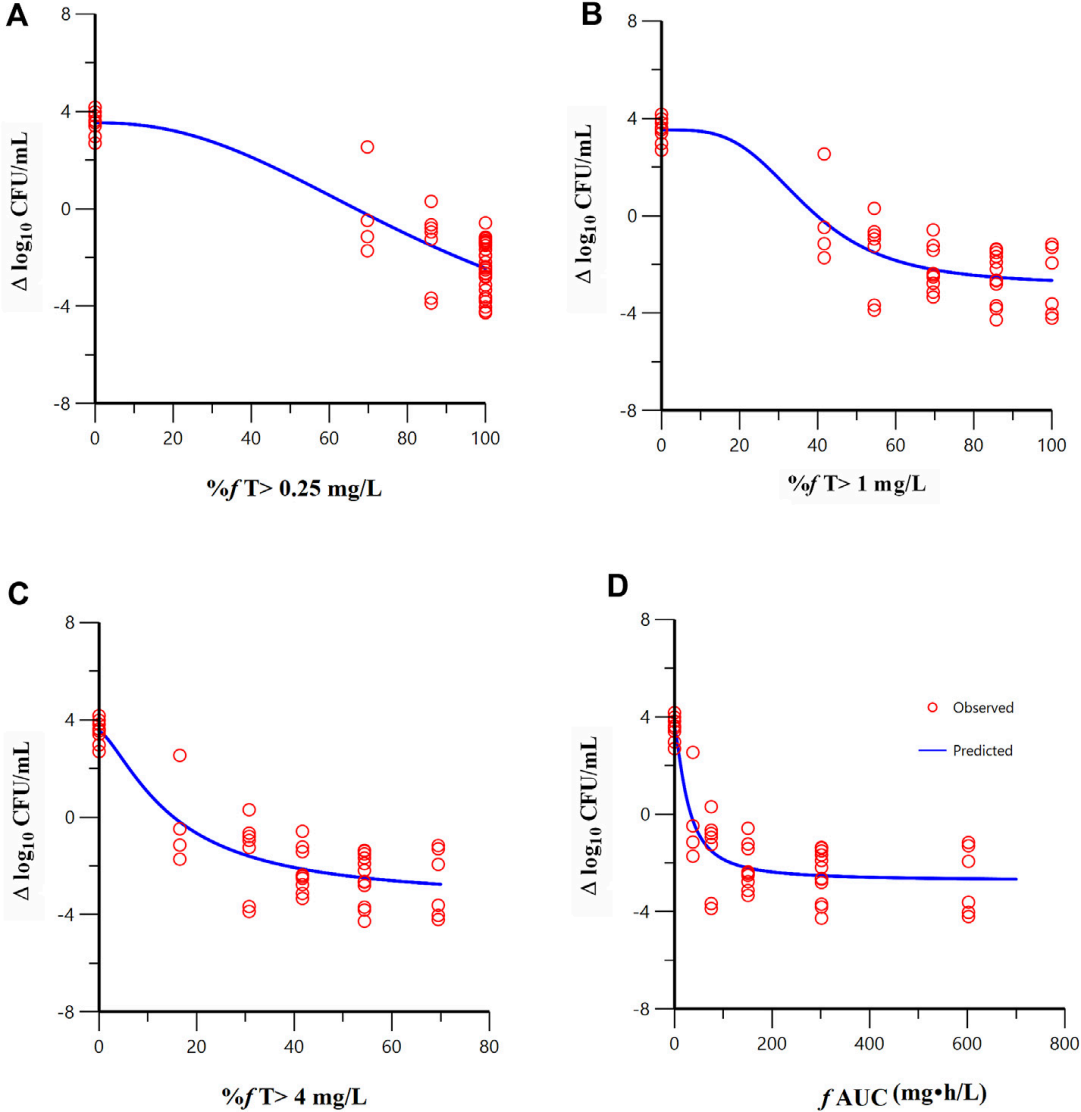
Relationships between the PK/PD indexes of FL058 in combination with meropenem and the change from baseline in colony count at 24 h. **(A)** %*f* T > 0.25 mg/L, percent time of the dosing interval that the free drug concentration is above 0.25 mg/L; **(B)** %*f* T > 1 mg/L; **(C)** %*f* T > 4 mg/L; **(D)** *f* AUC, area under the concentration–time curve from time 0 to 24 h, *f* denotes free drug. Open red circles denote observations; solid blue lines denote E_max_ model predictions.

FL058 cannot inhibit NDMs, which can hydrolyze meropenem rapidly (Ma et al., 2019). Therefore, the three strains of NDM-producing bacteria (ATCC BAA-2452, 17-R1-95, and 17-R2-27) were excluded from the PK/PD analysis. Sigmoid E_max_ model fitting results of the correlation between the PK/PD indexes of FL058 and change from baseline in colony count at 24 h in the *in vitro* model are shown in Figure 4. The estimated values of model parameters are shown in Table 3. The percent time of free drug above 1 mg/L (% *f*T > 1 mg/L), %*f*T > 4 mg/L, and area under the concentration–time curve from 0 to 24 h (*f*AUC) of FL058 showed a good fit (R = 0.896). With the smallest coefficient of variation (CV) of EC_50_ (the concentration of the drug that gives half-maximal response), %*f*T > 1 mg/L is the most efficacy-related PK/PD index of FL058. The PK/PD targets of FL058 are shown in Table 4. FL058 %*f*T >1 mg/L combined with meropenem achieved a static effect, and the 1- and 2-log_10_ kills in the colony count were 40, 48, and 64, respectively.

**TABLE 3:** E_max_ model estimates of the PK/PD indexes of FL058 and meropenem (CV%).

	PK/PD index		R		E_0_	E_max_		EC_50_	Γ	
FL058	%*f* T > 0.25 mg/L		0.894		3.53 (11.6)	11.04 (289.2)		92.88 (257.8)	2.28 (246.5)	
	%*f* T > 1 mg/L		0.896		3.53 (11.5)	6.38 (12.7)		37.52 (12.7)	3.55 (68.2)	
	%*f* T > 4 mg/L		0.896		3.53 (11.5)	6.99 (28.6)		15.11 (33.1)	1.42 (84.7)	
	*f* AUC		0.896		3.53 (11.5)	6.26 (10.2)		27.99 (34.6)	1.43 (60.5)	
Meropenem	%*f* T > MIC		0.931		3.34 (10.3)	6.07 (20.1)		71.70 (8.5)	6.18 (43.5)	
	*f* AUC/MIC		0.671		3.45 (20.8)	7.82 (>1,000)		1835.39 (1.3)	0.004 (>1,000)	
	*f* C_max_/MIC		0.671		3.45 (20.9)	7.81 (1.3)		996.22 (6.3)	4.07 (1.3)	

AUC, area under the concentration–time curve from 0 to 24 h; C_max_, maximum concentration; CV, coefficient of variation; E_0_, baseline effect; EC_50_, concentration of the drug that gives half-maximal response; E_max_, maximum effect; *f*, free drug; MIC, minimal inhibitory concentrationfor FL058 at 4 mg/L; PK/PD, pharmacokinetic/pharmacodynamic; γ, Hill coefficient; %*f* T > C_T_, the percent time of free drug above C_T_ (threshold concentration, C_T_: 0.25 mg/L, 1 mg/L, and 4 mg/L); %*f* T > MIC, the percent time of free drug above MIC.

**TABLE 4:** Targets of the PK/PD indexes for FL058 and meropenem.

	Static effect	1-Log_10_ kill	2-Log_10_ kill
FL058: %*f*T>1 mg/L			
Pooled analysis of seven strains	40	48	64
ATCC BAA-1705	30	39	52
17-R1-016	41	50	61
18-R1-38	54	60	89
Meropenem: %*f*T > MIC			
Pooled analysis of six strains	74	83	99
ATCC BAA-1705	95	98	no fit
17-R1-016	76	84	92
18-R1-38	79	88	97

**FIGURE 5:**
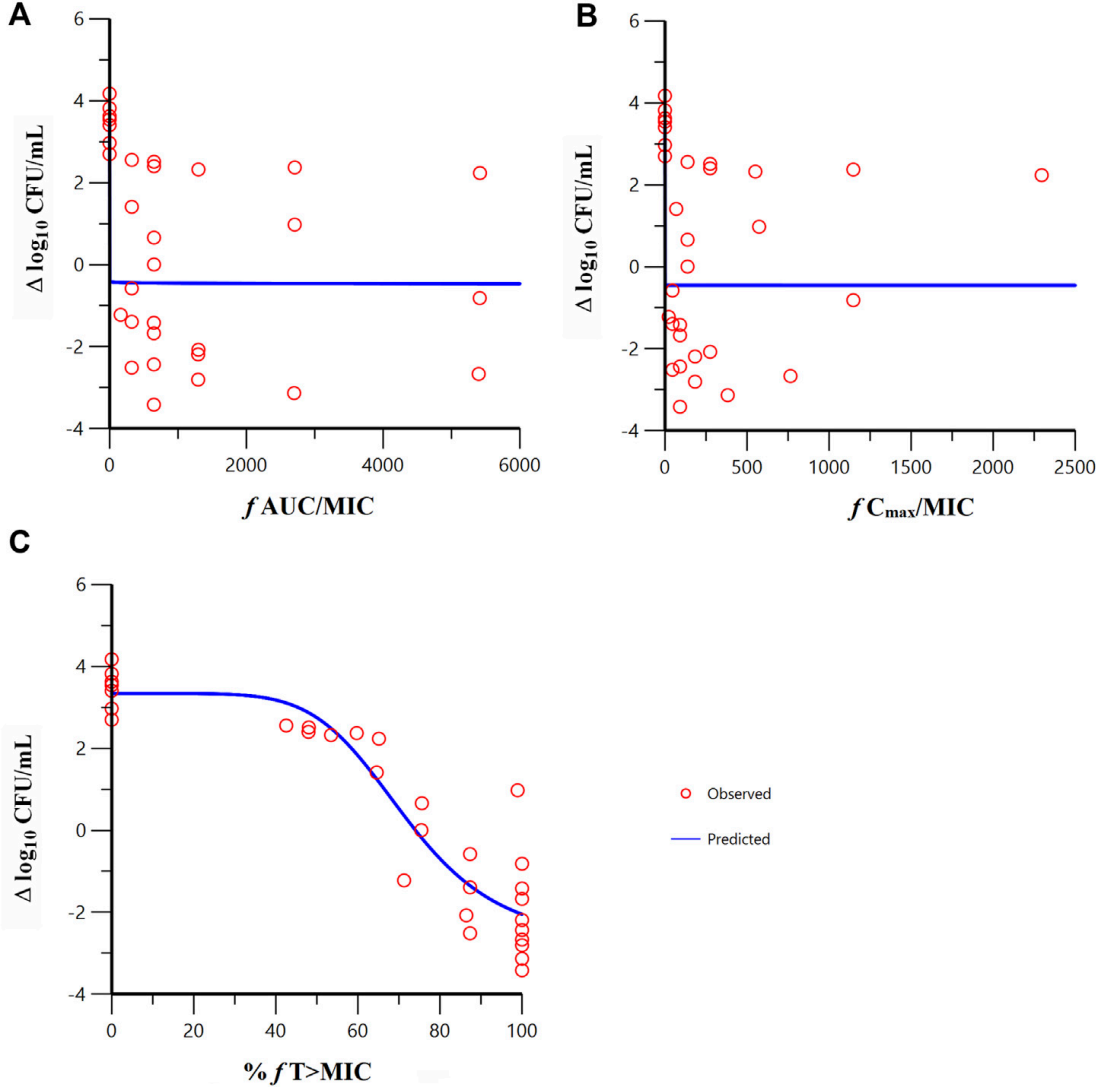
Relationships between the PK/PD indexes of meropenem in combination with FL058 and the change from baseline in colony count at 24 h. **(A)** *f* AUC/MIC, area under the concentration–time curve from 0 to 24 h over MIC; **(B)** *f* C_max_/MIC, maximum concentration over MIC; **(C)** %*f* T > MIC, percent time of the dosing interval that the free drug concentration is above MIC. *f* denotes free drug. Open red circles denote observations; solid blue lines denote E_max_ model predictions.

Sigmoid E_max_ model fitting results of the correlation between the PK/PD indexes of meropenem and change from baseline in colony count at 24 h in the *in vitro* model are shown in Figure 5. The estimated model parameter values are shown in Table 3. The best-fitting result was the percent time of free drug above MIC (%*f*T > MIC, MIC with FL058 at 4 mg/L) (R = 0.931), suggesting that the PK/PD index of meropenem remained %*f*T > MIC in combination with FL058 (Abdul-Aziz et al., 2015). The PK/PD targets of meropenem are shown in Table 4. %*f*T > MIC for meropenem combined with FL058 at a dose ratio of 2:1 achieved a static effect, and the 1- and 2-log_10_ kill in colony count values were 74, 83, and 99, respectively.

## Discussion

New beta-lactam antibiotic/beta-lactamase inhibitor combinations are being developed for clinical trials in response to the rapid global increase of beta-lactamase-producing Enterobacterales. It is necessary to fully understand the PK/PD characteristics of these combinations. The results from this study showed that for FL058 combined with meropenem, the best PK/PD index was %*f*T > C_T_, coinciding with avibactam. For relebactam and CB-618, which are structurally similar to FL058, the best PK/PD index was *f*AUC/MIC (Mavridou et al., 2015; Crass and Pai, 2019). The PK/PD index is %*f*T > C_T_ for beta-lactamase inhibitors of other structures such as tazobactam and enmetazobactam (Melchers et al., 2016; Bernhard et al., 2020), AUC for ANT2681 (Das et al., 2020), and *f*AUC/MIC for vaborbactam and taniborbactam (Griffith et al., 2019; Abdelraouf et al., 2020). More in-depth studies are needed to determine the best PK/PD index for FL058 in animal infection models or human patients.

In the *in vitro* model, FL058 0.5 g q8h infusion 2-h dose regimen had a %*f* T > 1 mg/L of 70%, which was greater than the target value of 64% needed for a 2-log_10_ kill. At an FL058 dosage greater than 0.5 g, the bacteria’s rebound growth at the trough concentration would be suppressed (Figure 2), suggesting the lowest effective dose (LED) of FL058 might be 0.5 g q8h infusion 2 h. This LED is on par with avibactam, a drug in the same class as FL058. The study data from meropenem administered at a daily dose of 3 g or 6 g (q8h) were subjected to pooled analysis to get the best PK/PD index and targets for FL058 because the grouped analysis of meropenem administered at a daily dose of 3 g was much closer to that of 6 g (Supplementary Figure S2). This finding coincides with the result that meropenem showed a comparable bactericidal effect at daily doses of 3 g and 6 g (Figure 2; Figure 3).

None of the currently market-available beta-lactam antibiotic/beta-lactamase inhibitor combinations can effectively treat NDM-producing Enterobacterales. Although ceftazidime/avibactam may be used in combination with aztreonam, the combination of these three drugs is of lower cost-effectiveness and higher safety concern. In contrast to avibactam and relebactam, FL058 alone in an *in vitro* susceptibility test showed some inhibitory activity against NDM-producing *E. coli* and, when used in combination with meropenem, had little inhibitory activity against NDM-producing *K. pneumoniae* (pending publication). In the *in vitro* model studies, however, FL058, in combination with meropenem, failed to suppress NDM-producing *K. pneumoniae* but did partially suppress NDM-producing *E. coli*. The possible reasons might be as follows: ① FL058 alone had some activity against *E. coli*, and its diazo heterocyclic ring structure was not hydrolyzed by NDMs, as shown in Supplementary Figure S3B. When *E. coli* ATCC BAA-2452 was cultured with FL058, the concentration of FL058 in the central compartment was consistent with the predicted concentration. ② Meropenem could be hydrolyzed by NDMs, but its concentration remained at a certain level in the central compartment where *E. coli* ATCC BAA-2452 was cultured, as shown in Supplementary Figure S3A. Moreover, the hydrolysis rate of beta-lactam antibiotics under the action of beta-lactamase had a positive correlation with the bacterial load (Kristoffersson et al., 2019). ③ When the two drugs were co-administered, their MIC against *E. coli* was low. ④ In the central compartment where NDM-producing *K. pneumoniae* was cultured, meropenem was almost completely hydrolyzed by NDMs, and its peak concentration was below the lower limit of detection. A current study of a mouse thigh infection model showed that FL058, in combination with meropenem at a level equivalent to 1 g/1 g q8h in humans, had close to a 2-log_10_ kill against NDM-producing *K. pneumoniae*. Meropenem has a broad antibacterial spectrum that covers multiple bacterial infections. Moreover, its bactericide activity against aerobic Gram-positive bacteria and anaerobic bacteria is significantly higher than that of aztreonam. Therefore, it is expected that this combination has a higher potential than the combination of aztreonam/avibactam, which is under development.

There were some limitations in this study. The killing curves of meropenem alone and FL058 alone against carbapenemase-producing Enterobacterales (CPE) were not assessed in the study because we believed it is unlikely that either of these monotherapies could be used for the treatment of an infection caused by CPE in clinical practice. The PK/PD index of meropenem was assessed when co-administered with FL058 in the ratio of 2:1, not in the ratio of 1:1. Subsequent studies will be carried out to further investigate the bactericide activity of FL058 in combination with meropenem against NDM-producing *E. coli*.

In summary, the best PK/PD index for FL058 in combination with meropenem in *vitro* model was %*f* T > 1 mg/L. The target of this index for achieving 2-log_10_ kill was 64%, which could provide a basis for assessment of the clinical dosing regimens of FL058 combined with meropenem. The lowest effective dose of meropenem/FL058 against KPC-/OXA-producing *K. pneumoniae* and *E. coli* was probably 1 g/0.5 g q8h infusion 2 h. Moreover, FL058 and meropenem might be a potential treatment for KPC- and/or OXA-48-producing Enterobacterales infection.

## Data Availability

The raw data supporting the conclusions of this article will be made available by the authors, without undue reservation.
