# Supplementary material for: In vitro pharmacokinetics/pharmacodynamics of FL058 (a novel beta-lactamase inhibitor) combined with meropenem against carbapenemase-producing Enterobacterales
Source: Front Pharmacol. 2024 Apr 11;15:1282480. doi: 10.3389/fphar.2024.1282480 (PMC11043595; doi:10.3389/fphar.2024.1282480)
Supplement: Supplementary file 1 [file DataSheet1.docx]

Supplementary Table 1 Dosing regimens of FL058 in combination with meropenem for pharmacokinetic/pharmacodynamic analysis

| Isolate no. | Dosing interval | Dosage (meropenem/FL058) |
| --- | --- | --- |
| FL058 pharmacokinetic/pharmacodynamic analysis | | |
| ATCC BAA-1705  17-R1-016  18-R1-38 | q8h | 1g/0.125g; 1g/0.25g; 1g/0.5g; 1g/1g; 2g/0.25g; 2g/0.5g; 2g/1g; 2g/2g;  blank control |
| 1. W45-56   20-W2-70  18-W2-18 |  | 1g/0.5g; 1g/1g;  2g/1g; 2g/2g;  blank control |
| 18-W1-29 |  | 1g/0.125g; 1g/0.25g |
| Meropenem pharmacokinetic/pharmacodynamic analysis | | |
| ATCC BAA-1705  17-R1-016  17-R1-38 | q8h | 1g/0.5g; 2g/1g |
|  | q12h | 1.5g/0.75g; 3g/1.5g |
|  | q24h | 3g/1.5g; 6g/3g |
|  |  | blank control |
| 18-W45-56  20-W2-70  18-W2-18 | q8h | 1g/0.5g; 2g/1g;  blank control |

2-h infusion


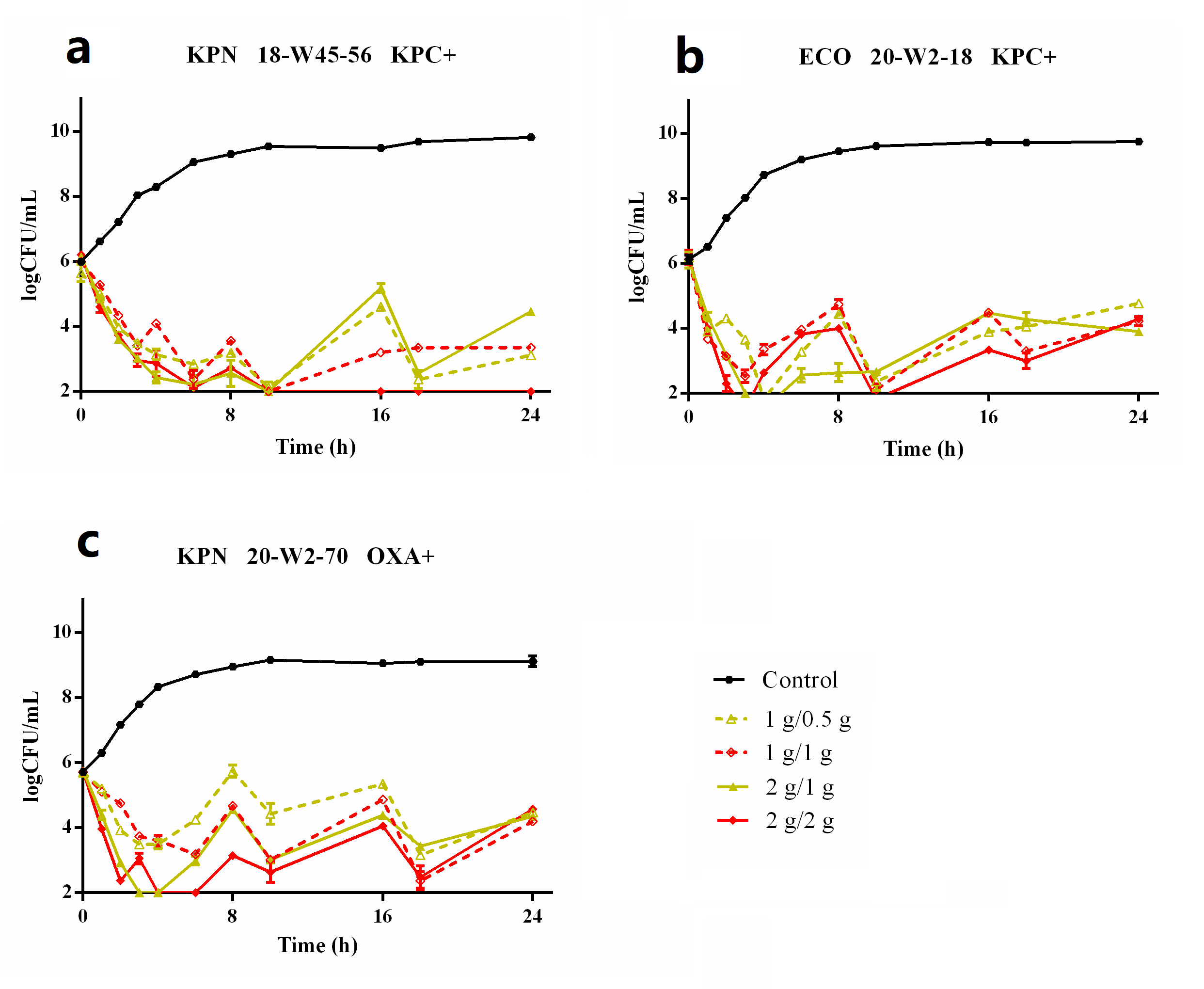


Supplementary Figure 1. Time-killing curves of meropenem in combination with FL058 q8h by a 2-h infusion against three KPC- or OXA-producing *K. pneumoniae* or *E. coli*. a. *K. pneumoniae* 18-W45-56; b. *E. coli* 20-W2-18; c. *K. pneumoniae* 20-W2-70. Tick marks on *x* axis are the start of each infusion. ECO *E. coli*; KPN *K. pneumoniae*.


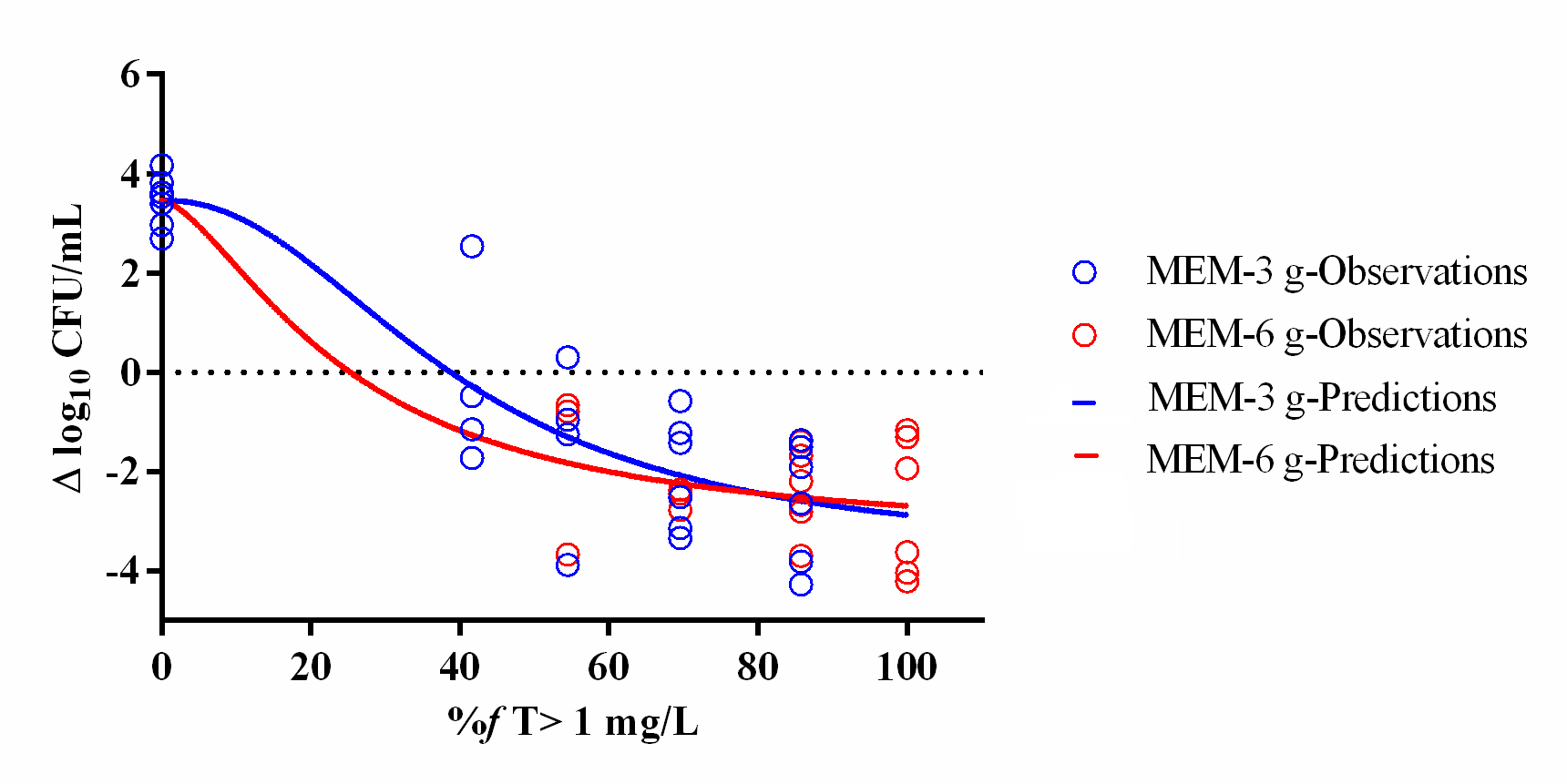


Supplementary Figure 2. Relationships between FL058 %*f*T>1 mg/L in combination with meropenem at daily dose of 3 g or 6 g and the change from baseline in colony count at 24 h. Blue open circles denote the observations from Meropenem Group 1 (daily dose=3 g), blue solid line for model predictions; red open circles denote observations from Meropenem Group 2 (daily dose=6 g), red solid line for model predictions.


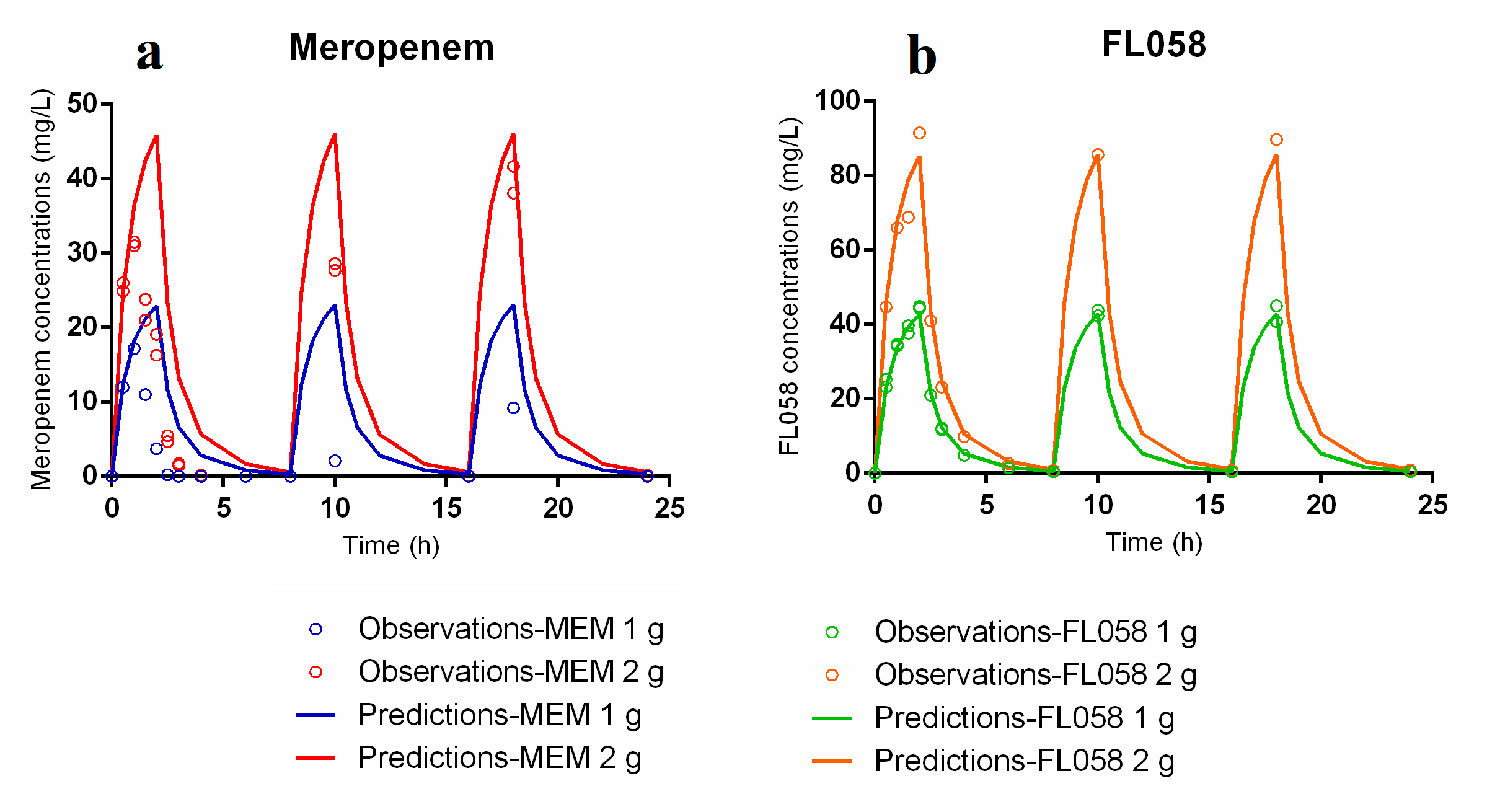


Supplementary Figure 3. Concentrations of meropenem (a) and FL058 (b) in central compartment vs. predictions when NDM-producing *E. coli* ATCC BAA-2452 was cultured.
